# Slower, shorter, sadder: a qualitative study exploring how dog walks change when the canine participant develops osteoarthritis

**DOI:** 10.1186/s12917-020-02293-8

**Published:** 2020-03-10

**Authors:** Zoe Belshaw, Rachel Dean, Lucy Asher

**Affiliations:** 1 PDSA Pet Hospital Nottingham, Dunkirk Road, Nottingham, NG7 2PH UK; 2 VetPartners, Leeman House, Station Business Park, Holgate Park Drive, York, YO26 4GB UK; 3 Newcastle University School of Natural and Environmental Science, Room 608, Agriculture Building, Kings Gate, Newcastle, NE1 7RU UK

**Keywords:** Dog walking, Osteoarthritis, Veterinary, Exercise, Qualitative, Social prescribing, Physical health, Canine

## Abstract

**Background:**

Dog walking may have multiple physical and mental health advantages, but not all dog owners appear to benefit. Dog health is a described barrier to dog walking activity, but specific causes and impacts of reduced exercise in owners of dogs with health problems have not previously been reported. The current study used a qualitative methodology to explore the impact of canine osteoarthritis on dog walking activity.

**Methods:**

Owners of dogs with osteoarthritis living in the United Kingdom (UK) were recruited through veterinary practices for semi-structured interview about life with an osteoarthritic dog. Participants were asked to reflect on walks that they had taken with their dog before he/she developed osteoarthritis, and to describe how those walks had changed. Interviews were transcribed verbatim and thematic analysis was used to construct key themes.

**Results:**

Forty owners of 35 osteoarthritic dogs were interviewed. Prior to their dog’s development of osteoarthritis, dog walking distance, speed and location were usually decided by the owner to satisfy the needs and enjoyment of dog and walker. A diagnosis of canine osteoarthritis led to both dogs and their owners altering the walks undertaken. Walks were typically slower, shorter and limited to locations where physical infrastructure, underfoot surface and gradients were perceived by the owner to be navigable by their dog. Most owners did not go on additional walks without their dog due to feelings of guilt and because walking without a dog was less enjoyable. Many owners described negative effects on their own physical health and diminished enjoyment of walking as a result of their dog’s condition.

**Conclusion:**

Our research suggests that osteoarthritic dogs may reduce the walking exercise their owners are able or willing to undertake. Since osteoarthritis is a common condition in older dogs, this is an important finding for those advocating dog ownership as a positive public health intervention. Strategies may be needed to ensure that owners of dogs that develop physical incapacities can continue to enjoy the health benefits they previously associated with dog walking. Future studies investigating dog walking activity should ensure that the health status of the dog has been considered.

## Background

Walking with a dog is a complex public activity that involves negotiation between dog and walker [1]. Dog walking research has predominantly focused on health gains associated with walking exercise. Benefits to owners’ physical and emotional health as a result of dog walking are well documented [2–9]. A meta-analysis of dog walking literature [10] identified that dog owners walk for more minutes per week than non-dog owners, and that acquisition of a dog could lead to a sustained increase in physical exercise. Subsequent research with similar findings has led some authors to suggest dog ownership, and dog walking, could be a positive public health intervention to tackle human obesity [5, 7, 8] and to improve physical and mental health [9]. Around 50% of dogs visiting veterinary practices in the UK dogs are currently estimated to be overweight or obese [11], and there is an ongoing dog overpopulation problem [12]. Therefore, an increase in dog ownership and dog walking could also have dog welfare implications.

However, the same meta-analysis [10] demonstrated that not all dog owners walked further than non-owners. Environmental and psychological motivators associated with dog walking include: a sense of obligation; support and motivation provided by a dog; an accessible, pleasant, safe and interesting environment; a desire to keep fit or lose weight; and positive interactions with other owners and their dogs [1, 4, 6]. Barriers may include: local legislation on how and where dogs can be exercised; ownership of a smaller, old, ill or unsociable dog; adverse weather; the owner’s health state; and poor relationships with others using the same dog walking spaces [1, 4]. Several studies [1, 4, 13, 14] also point to the importance for the owner that they perceive their dog to be enjoying their walk.

Whilst the dog’s health has been identified as a barrier to dog walking in several studies, its impacts on the dog walker have yet to be explored in detail. Osteoarthritis is estimated to affect the joints of 2.5% of all dogs in the United Kingdom [15]. It causes stiffness and pain which may directly impact the dog’s desire, and ability, to walk [16]. Owners of osteoarthritic dogs may be advised by veterinary surgeons to limit their dogs’ exercise to help manage their condition [17]. However, the impact of a change in the dog’s orthopaedic health status on their owners’ walking behaviour has not been investigated. Such research should enable stakeholders including veterinary surgeons, owners, and public health policy makers to better understand the complexities of interaction between dog and owner during walking exercise.

This study therefore aimed to answer the research question: how are dog walks affected when a dog develops osteoarthritis? The objective was to conduct semi-structured interviews with owners of osteoarthritic dogs to understand how dog walks changed before and after the diagnosis.

## Results

Fifty-eight owners of osteoarthritic dogs expressed interest in participation. Fifteen subsequently declined to be interviewed, five were unavailable during the study period, four expressed interest only after the study had closed due to data saturation being reached, and two dogs were euthanized before the interviews with their owners could take place. Thirty-two interviews were conducted, involving 40 participants who discussed managing 35 osteoarthritic dogs. Male and female participants of a range of ages and backgrounds were recruited from the rural Westcountry to inner city Scotland and fulfilled all aspects of the sampling frame (Supplementary Data 1, Additional file 1). Interviews ranged from 52 to 170 min in duration. Four themes were constructed. The data below comprise a subtheme of the theme relating to the impact on owners of their dog developing osteoarthritis. Illustrative exemplary quotes are included. Where more than one person was involved in an interview, their quotes are identified as interviewee a or b, as determined by in the order in which they first spoke during the interview.

### How dogs were walked before developing osteoarthritis

Almost all dogs had a single primary walker who took them on most of their walks. Many owners described a long-standing enjoyment of walking, but a few recalled that they had not liked walking until they acquired a dog. For many, watching their dog having fun enhanced the pleasure of walks, and dogs often acted as a gateway to increased social contact with other walkers. Acquiring a dog had provided several male owners with a perceived legitimacy to walk in green public spaces and had helped a wheelchair-bound man integrate in the community.

*You can’t help people. Guys walking in a park, it’s just not right. And I was walking round the park one day, I saw a guy with his dog one day, and I thought ‘I need to get a dog.’ I love having a dog, and I love going for walks. So I feel better with a dog*. [Interview 26].

*My dad was in a wheelchair, but he still managed to take [dog's name] out first thing in the morning. And my dad ended up talking to everybody in the street because [dog's name] got him out. Granted it was only round the area, but he ended up meeting everybody and talking to people, particularly in the summer.* [Interview 31].

Most dogs received two broad, overlapping, categories of walks, which we will term “functional” and “leisure”. “Functional” walks had the primary objective of fitting the dog’s needs to toilet and exercise around their owner’s available time. These were short, local-to-home walks that varied little, and were typically conducted one or twice daily by the primary walker independent of weather or season. Descriptions of these walks often framed them in terms of duty and necessity though some reflected that these walks were useful to make the owner exercise when they might not otherwise do so, particularly in poor weather.

*I walk the dogs, only for ten, 15 min, I don’t have more time, during lunch, and it’s not at a set time point. Sometimes it’s at twelve, somewhere between twelve and three o’clock.* [Interview 19].

*Having a dog does focus you …*. *that you have to do it … ..* [Interview 15, interviewee 15b].

“Leisure” walks were usually longer in length, less time pressured and typically occurred in green or rural spaces, sometimes a distance from home. Their location and timing could vary spontaneously in response to daylight, season, weather, the owners’ mood and whether other family members wanted to participate. In this context, owners described walking *with* the dog, not *for* the dog. Working owners typically conducted these walks in the evenings, at weekends or on holidays whilst those who did not work usually had less time restriction on their occurrence. Leisure walks often replaced at least one functional walk that would otherwise have occurred on the same day. Several owners described choosing holiday locations specifically based on day-long leisure walks they could enjoy with their dog.

17a *I suppose the typical walk would be a mile. But then you could do much longer...*

17b *Oh, they could do much longer than that.*

17a *They did ten or twelve. [Dog’s name] did eighteen once, didn’t he?* [Interview 17].

Benefits of leisure walks appeared diverse and multi-faceted, permitting both dog and owner to relax and enjoy their surroundings. Whilst leisure walks were often chosen on the basis of a landscape or route owners wished to visit, some also consciously chose locations where their dog would be able to exercise off lead and play. Pleasure for many owners was clearly derived from watching their dog have fun. Whilst a few actively sought solitude during their walks, many described enjoyable interactions with other walkers in green spaces. Several had developed new friendships as a result. Rarely, owners felt excluded from joining existing social groups of dog walkers if their dog did not fit in, typically due to its behaviour.

*You meet all sorts of people. And if you’ve got a dog, they’re happy to talk….* [Interview 12].

*So I started going out every morning once I’d dropped the kids off at school. And there’s loads of dogs, they meet every morning, that’s a wee clique. And I’d say to them “He’s no good with other dogs.” And they’re “Oh, it’s fine, it’s fine”. Until he went “Rrrrr” at one of the women…..* [Interview 29].

### Why dog walks changed when the dogs developed osteoarthritis

All owners described a change to their walks following their dogs’ diagnosis with osteoarthritis. Many recalled advice from their veterinary surgeon to reduce their dog’s walk length, to keep their dog on lead exercise only and/or to keep exercise levels consistent day-to-day. Many had tried to adhere to this advice, but some found it impractical.

*My vet was very clear about it. You walk him for 10 min, 10 min maximum. Ten minutes with him, he stops at every gatepost, at every twig. Ten minutes is about fifty yards!* [Interview 12, interviewee 12a].

Commonly, the dogs had also changed the nature of their own walks by walking more slowly and stopping more frequently, usually to sniff but sometimes apparently to rest. Some sat down or looked at their owners when confronted with large hills, rough terrain or after a certain distance or duration. Typically, owners took this as the signal that the dog had had enough and truncated the walk. Dogs were observed to have both good and bad days which impacted on their desire, and ability, to exercise. Some owners had restricted their dogs’ walk length or stopped engaging them in active play to reduce their stiffness the following day, despite sometimes knowing the dog would exercise more if permitted. Several described making daily assessments of their dog’s gait and attitude to decide how far, if at all, to walk them that day. Rarely, owners were keen that their dog went for a walk regardless of their willingness to do so.

*I think that one walk a day, and if she can do a mile, then, I wouldn’t like to say much more than a mile, I think some of the walks are a-mile-and-a-third, but that’s the limit. But I think she seems better for it, and then she probably comes back and she sleeps more soundly.* [Int 15, interviewee 15b].

### How dog walks changed after the diagnosis

Osteoarthritis led to changes in the length, speed, duration and location of walks. One very severely affected dog was no longer walked, and several large breed dogs were unable to easily get into vehicles so could not be taken on leisure walks. For others, shorter functional and leisure walks were still possible. However, every owner of an older affected dog, independent of their breed or previous behaviour on walks, described their dog’s increasing tendency to walk more slowly, cover less distance and spend more time sniffing. All thought they were walking significantly less far with their dog than before it developed osteoarthritis. Several expressed great frustration at the slow pace at which their dogs’ walks now proceeded, and sniffing behaviour led some owners to continue to walk their dogs off the lead despite receiving veterinary advice to the contrary. Typically, owners walked ahead at their own pace for a few minutes, then stopped and waited for their dog to catch up.

*It’s the way she’s slowed down. She does more sniffing than walking, yes. Yeah, that was one of the significant things that, as she slowed down there was more and more of this sniffing… At first it was a massive pain, very frustrating.* [Interview 15, interviewee 15b].

*She knows the parks, we go to these big parks all the time, and we get out the car, she knows which way to go, she goes the route. And I’ve just noticed, the last few months, rather than going the one way she always wants to go, she’s now cutting a wee corner off here, a wee corner off there, as if just to get herself back. She knows her limitations.* [Interview 26].

Owners of more than one dog described challenges of combining the exercise needs of affected and unaffected dogs. Some took their dogs on separate functional walks, others chose leisure walks where the osteoarthritic dog could take short-cuts or could sit and wait. Rarely, owners had adapted pushchairs, prams or even a wheelbarrow to make multi-dog walks easier.

*I’ve taken her in the wheelbarrow for a long walk, but it was downhill and it was bumpy, and it’s hard work. But going back up the hill with 32 kilos of dog that got up to look and move was really hard work.* [Interview 16].

*If we take them out in the woods, you’re trying to keep your eye on [unaffected dog's name] because she’s a long way ahead, or can be, and this one [affected dog's name] is trailing behind….* [Interview 15, interviewee 15a].

Owners described feeling heightened responsibility and less spontaneity when walking an osteoarthritic dog. Many discussed the need to consider hazards such as walls, steps, bridges and stiles that their dog might encounter, and had learnt to plan routes to avoid these, or had developed novel strategies to cope. This was particularly difficult for older owners, owners of larger dogs and those living in rural areas. A few owners expressed concern that people might think they were being cruel walking a stiff, slow dog down the road, and avoided certain routes for this reason. Conversely, owners of younger dogs described facing difficult comments from other owners about their apparent lack of exercise.

*Some stiles are quite high. And I’m thinking ‘Hold on, I’ve got to pick him up.’ and then when you lower him down on the other side… Yeah, to be quite honest, I pick him up by his tail and his collar. Just pick him up, get him as low down on the other side before you let him go.* [Interview 24, interviewee 24a].

*People say ‘You don’t walk your dog very far.’ Yeah, but it means she can keep walking. So we have to accept that other people with Labradors of that age can do miles and miles of walks every day. She can’t do that.* [Interview 23].

Almost all owners said they would not go for a leisure walk without their dog. Reasons included guilt at leaving the dog behind, feeling that a walk was not the same without their dog by their side, and not being able to find someone to look after the dog while they went out for the day. Several owners said they had gained weight since their dog developed osteoarthritis and reflected that they had not realised how much exercise they had been deriving from dog walking. Consistently for male owners, there was a sense that without their dog they were perceived to be a threat by some women if walking in a rural area. This acted as an additional barrier to them going on rural leisure walks without a dog.

*I’ve said to people, ‘I don’t walk as far with my dog, I’m putting weight on,’ in conversation, not as a moan. No, I don’t take him as far.* [Interview 2].

*Oh, no. I couldn’t go off and leave him here and go for a long walk. I’d feel guilty actually. No, I’d rather do more, shorter walks and let him come with me.* [Interview 5].

*It’s one of those, it’s a middle-aged man walking on his own is quite... You can see people going ‘What are you doing here?’ It’s a little bit odd. If you’re out in the woods, out in the fields, a guy on his own is a bit strange. You’ve got a dog with you, and it’s ‘Morning! How are you?’* [Interview 11].

## Discussion

This qualitative research is the first to indicate that an osteoarthritic dog may limit the owner’s dog walking exercise and reduce the health benefits of dog ownership. These data also demonstrate how owners’ emotional attachment to their dogs may act as both a motivator and a barrier to dog walking exercise. These findings have important implications for those advocating dog ownership or dog walking as a route to improved public health [5, 7–9]; those benefits may be negated when the dog becomes physically incapacitated. Osteoarthritis is just one example of a canine health problem that limits mobility, and it is likely that a wide range of other prevalent canine conditions including cardiac disease [18] and obesity [19, 20] will have a similar impact. As such, these data may represent a common but previously undescribed aspect of dog walking exercise. The physical, and perhaps behavioural, health status of the dog being walked should be taken into account in future research on dog walking.

It is important to note the limitations of this study so its significance can be correctly interpreted. As this qualitative research involved a tiny fraction of the estimated 9.4 million dogs in the UK [21], these data cannot be taken to be representative of all walkers of osteoarthritic dogs, and cannot be used to infer the frequency with which these experiences might occur. However, all interviewees described a change in their dog walking behaviour after their dog developed osteoarthritis. It is therefore likely that a dog’s physical health is an important factor in dog walking exercise. Interviews relied on owners’ recall of their previous walking behaviour which may have been incorrect or biased, but the only alternative to understand how walking exercise changed would be a logistically challenging long-term cohort study. Despite these limitations, this first report of the impact on dog walking of poor canine physical health provides an important contribution to the literature.

These data build on a growing body of qualitative research describing walks with healthy dogs [4, 7, 13]. Previous studies have identified the primary motivations for dog walks as meeting the dog’s physiological or health need to walk [4, 13, 22] and/or because walking the dog makes the owner happy, contingent on the dog also enjoying the experience [13]. Our data suggest different walks undertaken by the same healthy dog-owner pair may have different motivations; reasons for, and benefits from, “walking the dog” versus “walking *with* the dog” may be usefully explored in future research. Our data also confirm that, as identified by Westgarth [13], dog owners may not identify dog walking as a significant form of exercise until they are no longer able to perform the activity. Westgarth [13] did not identify a difference between dog walker gender in walking motivation or behaviour. In contrast, male interviewees in this study presented the dog as an important facilitator for them to be able to access to green spaces without being perceived as a threat to women. Given the drive to increase walking exercise for public health reasons [6], this warrants further, specific investigation.

Little has previously been described about the challenges associated with walking dogs with health problems, perhaps because the dogs included in most previous research were presumed to be healthy. Ill dogs, old dogs and multiple dogs were identified as barriers to dog walking in a survey of 431 predominantly Caucasian, middle aged, female dog owners in the USA [14] but the reason why these are barriers had not been explored in detail. Our research suggests that a dog’s physical health problem may limit both physical and mental health benefits associated with dog walking through slower, shorter, less varied and less relaxing walks.

Whilst owners may set the agenda for the walk destination and distance with a healthy dog, an osteoarthritic dog’s needs appear to dominate decision making about their walk location, speed and distance, perhaps to the detriment of their owner’s enjoyment. This is an important, novel finding with significant implications for those interested in promoting dog walking activity. Some owners modified their walking behaviour on the basis of veterinary advice, but the evidence base for such recommendations is weak. Veterinary surgeons may be unaware that recommendations to limit a dog’s exercise may be detrimental to the owner, and may also compromise the welfare of the dog through boredom [23] and an increased risk of obesity which in turn may further decrease their exercise capacity. These data suggest collaboration is needed between veterinary surgeons, healthcare professionals and those interested in promoting walking exercise as a public health intervention to fully understand the motivators and barriers to this form of exercise. Collection of quantitative accelerometry data, including distance travelled and gait parameters, from affected dogs during walks is likely to be useful to further inform this discussion [24–27]. Such data could also be important to owners and veterinary surgeons in monitoring individual dogs’ disease progression, and the welfare impact of any interventions.

The implications of owners’ emotional attachment to their dogs were evident in this study as a motivator to walk [13] but importantly also as a barrier. Just as owners in Westgarth’s [13] study described guilt at not taking their dogs for a walk, owners in the current research articulated guilt associated with leaving the dog behind. Dogs may survive for several years with health conditions that restrict how they can be exercised [15, 18], and some dogs in the current study were diagnosed with osteoarthritis as young adults with their walking exercise impaired the rest of their lives. It is therefore important for advocates of dog ownership for human health to understand the strength and importance of the dog-owner emotional bond and its capacity to limit as well as promote dog walking exercise. If dog ownership is to be advocated, strategies should also be developed to support owners of dogs who can no longer exercise their dogs without restriction.

## Conclusions

Dogs walks may be subdivided into those primarily taken to meet the physiological needs of dog, and others primarily for the enjoyment of dog and owner. Canine osteoarthritis may lead to significant decreases in the distance, duration and pace of walks, and may diminish their owners’ enjoyment of walking activity. Future work should explore other canine health problems to determine whether they similarly impact on owners’ walking exercise. These findings have important implications for policymakers advocating dog ownership as a means of improving walking activity. Interventions to sustain walking exercise among owners of dogs with physical health problems may be required, and the health status of dogs should be included in future dog walking research.

## Methods

Data presented are taken from a rich qualitative study using interviews and focus groups to explore the experiences of dog owners, veterinary surgeons and veterinary nurses who manage dogs with osteoarthritis. Some results and methodological details have previously been reported [28]. Reporting follows the Consolidated Criteria for Reporting Qualitative Research (COREQ) [29].

### Interview process

The inclusion criteria for interviewees were: a) ownership of a dog at least 5 years of age treated or managed for osteoarthritis in at least one limb; AND b) residency of dog and owner(s) in the UK. Recruitment was based on a purposive sampling frame constructed by the authors (Supplementary Data 2, Additional file 1) intended to capture the widest possible range of owner experiences.

Most interviewees were recruited by displaying information posters in a convenience sample of 10 veterinary practices in England and Scotland. Other interviewees were recruited by snowball sampling or from the authors’ networks. Incentives to participate were not provided. Interested owners were sent information about the purpose of the study including details of the interviewer (ZB)’s background as a veterinary surgeon and previous owner of an osteoarthritic dog. If they were willing and eligible to participate, an interview date was arranged. All interviews were conducted face-to-face by ZB in owners’ homes between February and August 2014. A semi-structured interview guide, piloted with eligible owners before use (Supplementary Data 3, Additional file 1), covered topics ranging from the owner's acquisition of, and relationship with, their dog to the way treatment decisions were made. The guide was used to ensure broad topics were not missed but owners were encouraged to lead the interview. All family members who had a direct role in caring for an eligible dog were invited to participate in the same interview, and all eligible dogs within each household were discussed. Written consent to participate, and for anonymised data to be used in publications, was obtained from each interviewee. Pertinent to this publication, interviews explored owners’ experiences of dog walking, and how those walks had changed since their dog’s diagnosis with osteoarthritis.

### Thematic analysis

Interviews were audio recorded and professionally transcribed intelligent verbatim. Transcribed interviews were reviewed several times by the lead author in combination with contextual field notes made during the interviews and focus groups. Transcripts were checked for accuracy against the audio recording but were not returned to interviewees. Thematic analysis was performed by ZB with assistance from the other authors, following the six step plan described by Braun and Clarke [30] using the organisational support of nVivo (nVivo v10, QSR). Analysis was performed in parallel with data collection; constant comparison was used to ensure all opinions were included [30, 31] and themes were inductively identified from semantic and latent codes. Data saturation was defined as the point at which no additional themes emerged as a result of analysing new transcripts; interview recruitment was stopped at this point. Statistical analysis was not performed as the qualitative purposive sampling methodology aimed to capture a wide range of experiences rather than to represent a population [32, 33]. The number of participants included was considered sufficient for this exploratory research given the tight inclusion criteria, in-depth nature of the interviews, the personal experience of the interviewer in the situation discussed and the cross-case analysis performed [34].

## Supplementary information

**Additional file 1: Supplementary Data 1.** Summary of the interview participants’ coverage of the sampling frame; tabulated data of participant numbers in each of the sampling frame categories. **Supplementary Data 2.** Purposive sampling frame used to recruit dog owners to the study; complete sampling frame including response options that was constructed to ensure a broad range of participants were included in the interviews. **Supplementary Data 3.** Interview guide used during semi-structured interviews; complete semi-structured interview schedule including prompt questions.

## Data Availability

The datasets generated and/or analysed during the current study are not publicly available due as they contain information that could compromise participant privacy and consent, but are available from the corresponding author on reasonable request.

## Abbreviations

COREQ: Consolidated Critiera for Reporting Qualitative Research
UK: United Kingdom
